# The ARSQ 2.0 reveals age and personality effects on mind-wandering experiences

**DOI:** 10.3389/fpsyg.2014.00271

**Published:** 2014-04-03

**Authors:** B. Alexander Diaz, Sophie Van Der Sluis, Jeroen S. Benjamins, Diederick Stoffers, Richard Hardstone, Huibert D. Mansvelder, Eus J. W. Van Someren, Klaus Linkenkaer-Hansen

**Affiliations:** ^1^Department of Integrative Neurophysiology, Center for Neurogenomics and Cognitive Research, VU University AmsterdamAmsterdam, Netherlands; ^2^Neuroscience Campus AmsterdamAmsterdam, Netherlands; ^3^Department of Complex Trait Genetics, Center for Neurogenomics and Cognitive Research, VU University Amsterdam and VU Medical Center AmsterdamAmsterdam, Netherlands; ^4^Department of Sleep and Cognition,Netherlands Institute for NeuroscienceAmsterdam, Netherlands; ^5^Department of Medical Psychology, VU University Medical CenterAmsterdam, Netherlands

**Keywords:** Amsterdam Resting-State Questionnaire (ARSQ), consciousness, mind wandering, personality traits, test-retest reliability

## Abstract

The human brain frequently generates thoughts and feelings detached from environmental demands. Investigating the rich repertoire of these mind-wandering experiences is challenging, as it depends on introspection and mapping its content requires an unknown number of dimensions. We recently developed a retrospective self-report questionnaire—the Amsterdam Resting-State Questionnaire (ARSQ)—which quantifies mind wandering along seven dimensions: “Discontinuity of Mind,” “Theory of Mind,” “Self,” “Planning,” “Sleepiness,” “Comfort,” and “Somatic Awareness.” Here, we show using confirmatory factor analysis that the ARSQ can be simplified by standardizing the number of items per factor and extending it to a 10-dimensional model, adding “Health Concern,” “Visual Thought,” and “Verbal Thought.” We will refer to this extended ARSQ as the “ARSQ 2.0.” Testing for effects of age and gender revealed no main effect for gender, yet a moderate and significant negative effect for age on the dimensions of “Self,” “Planning,” and “Visual Thought.” Interestingly, we observed stable and significant test-retest correlations across measurement intervals of 3–32 months except for “Sleepiness” and “Health Concern.” To investigate whether this stability could be related to personality traits, we correlated ARSQ scores to proxy measures of Cloninger's Temperament and Character Inventory, revealing multiple significant associations for the trait “Self-Directedness.” Other traits correlated to specific ARSQ dimensions, e.g., a negative association between “Harm Avoidance” and “Comfort.” Together, our results suggest that the ARSQ 2.0 is a promising instrument for quantitative studies on mind wandering and its relation to other psychological or physiological phenomena.

## Introduction

Recent estimates suggest that the human brain engages in mind wandering for approximately half of its waking day, thereby generating thoughts and feelings unrelated to current external demands (Killingsworth and Gilbert, 2010). Despite the ubiquity of mind wandering, research into its nature has since the 1970's (Antrobus, 1968; Antrobus et al., 1970; Singer, 1974) received limited attention until recently reinvigorated (for a review, see Smallwood and Schooler, 2006). The state of wakeful rest—or “resting state”—serves a special role in this context, as it is both frequently employed during functional neuroimaging and may be viewed as a model system to study mind wandering relatively free from external demands.

Most investigations of mind wandering have utilized task-based designs (Antrobus et al., 1966; Christoff et al., 2009; Schooler et al., 2011), enabling the detection of mind wandering episodes as a function of task parameters (e.g., difficulty). Yet, our understanding of the content of mind wandering has remained limited possibly due to lack of established instruments and protocols (for pioneering efforts, see Lehmann et al., 1995; Andrews-Hanna et al., 2010; Delamillieure et al., 2010). In an attempt to capture mind-wandering experiences during the resting state in a standardized fashion, we recently presented the Amsterdam Resting-State Questionnaire (ARSQ) as an efficient self-report tool (Diaz et al., 2013). The ARSQ facilitates a quantitative assessment of thoughts and feelings along several dimensions of mind wandering obtained through factor analysis techniques. This has paved the way for investigating associations between mind-wandering experiences and psychological or physiological variables such as mental health (Diaz et al., 2013), but also gender, age, or personality.

Mind wandering may intuitively seem involuntary and unrestrained in nature. However, recent data from our experiments indicated that ARSQ scores remain significantly correlated between assessments almost 1 h apart (Diaz et al., 2013). This observation raised the question as to what extent mind wandering is state-like (e.g., dependent on situational factors) or trait-like, i.e., partly reflecting stable individual differences. Personality traits could be potential contributors to this stability in mind wandering, considering the DSM-IV definition of a personality disorder and its link to inner experience: *“An enduring pattern of inner experience and behavior that deviates markedly from the expectations of the individual's culture”* (American Psychiatric Association, 2000). Cloninger's Temperament and Character Inventory (TCI, Cloninger et al., 1993) is an established instrument to quantify personality traits along the dimensions of a psychobiological model. Crucially, this psychobiological model distinguishes between subconscious “temperaments” and conscious “characters.” Temperaments are defined as primarily engaging perceptual memory systems, i.e., automatic responses to stimuli and are categorized as “novelty seeking,” “harm avoidance,” “reward dependence,” and “persistence” (Cloninger et al., 1993). Characters by contrast, are related to different conscious concepts of self and are reliant on declarative memory systems (Cloninger et al., 1993; De Fruyt et al., 2000; Watson and Tharp, 2014), e.g., verbal/visual imagination and symbolic reasoning fall in the categories “self-directedness,” “cooperativeness,” and “self-transcendence.” It is precisely these conscious, self-oriented experiences associated with these character dimensions that we expected to exhibit the strongest overlap with mind wandering, itself defined as conscious thought and feelings unrelated to an external task (Smallwood and Schooler, 2006; McVay and Kane, 2010).

In the present study, we first developed an improved extended 10-dimensional model of resting-state experiences, further increasing practical utility by standardizing the number of items per dimension and allowing for the quantification of more qualitative aspects of mind wandering such as visual and verbal thought. This updated model was subsequently used to test for gender and age specific effects on mind-wandering experience, test-retest stability over time-scales up to 32 months between assessments and, finally, the relationship between ARSQ and personality traits of the psychobiological model.

## Materials and methods

### Participants

Data were obtained from the Netherlands Sleep Registry (NSR, www.sleepregistry.org), a database aimed at sampling multiple questionnaires in a large cohort comprising the full range from very light to very sound sleepers. Registered participants were invited to complete the Amsterdam Resting-State Questionnaire (ARSQ) and a variety of psychometric instruments (see below) using their home computer. All instruments, including the ARSQ, were administered in Dutch. This yielded two large samples of participants filling out the 50-item ARSQ (*n* = 882, 70% females, age range 19–85, mean age 53.9) as described recently (Diaz et al., 2013), and an extended 54-item version (*n* = 562, 76% females, age range 20–86, mean age 54.4). The study protocols presented here were approved by the institutional review board of the VU University Medical Centre, Amsterdam, The Netherlands.

### Online ARSQ assessment procedure

Participants were first (screen 1) asked to enable and test their PC audio equipment (i.e., turn on speakers or put on headphones) and afterwards proceed to the next screen. The following screen (screen 2) started with the following instruction (translated from Dutch): *“A rest period of five minutes will shortly start. It is important that you relax and remain seated quietly behind your computer with your eyes closed. Try not to fall asleep. Instruction: Once you click on ‘next,’ a timer will count down from 5 minutes to 0. Once the resting period is over, you will hear a beep. Subsequently you will open your eyes and follow the instructions. Should you be interrupted during the 5 minutes by something or someone, you can open your eyes and click on ‘Stop’ and subsequently on ‘Restart’ to newly start the 5 minutes rest session. Now, sit down quietly, click ‘next’ and immediately close your eyes to start the resting period.”* The next screen (screen 3), triggered by initiating the experiment, showed the following text: *“If the resting period has ended you will hear a beep. You can stop the beep by clicking ‘Stop’. Afterwards click on “next” to proceed to the questions.”* Subsequently (screen 4), the participant was briefly informed about how to rate the questions: *“The 5 minutes of rest are over. Now several statements will follow regarding potential feelings and thoughts you may have experienced during the resting period. Please indicate the extent to which you agree with each statement.”*

In order to identify invalid trials, participants in the NSR sample indicated at the end of the questionnaire whether the eyes-closed rest session was interrupted or not, with the option to give a detailed reason. The item order for the extended ARSQ was randomized, except for the last two validation items (“I had my eyes closed” and “I was able to rate the statements). All statements were scored on a five-point Likert-type scale (1–5) with the labels “Completely Disagree,” “Disagree,” “Neither Agree nor Disagree,” “Agree,” and “Completely Agree.”

### International personality item pool

A set of 137 personality items (see Table S1 and http://ipip.ori.org) obtained from the international personality item pool (IPIP, Goldberg, 1999; Goldberg et al., 2006) was included and rated “yes” or “no” by a subset of the participants (*n* = 502, 78% female, mean age 54.6). These items are proxy-measures of Cloninger's Temperament and Character Inventory (TCI, Cloninger et al., 1993), quantifying the temperaments “Novelty Seeking,” “Harm Avoidance,” “Reward Dependence,” “Persistence” and characters “Self-directedness,” “Cooperativeness,” and “Self-Transcendence.” The choice for this set of IPIP items for use in the online test battery of the NSR was primarily motivated by the significant relationship between sleep disorders and temperaments and characters (De Saint Hilaire et al., 2005; An et al., 2012). In addition, the IPIP proxy-measures of Cloninger's TCI exhibit both a high mean correlation of 0.86 with the original scale as well as near identical mean reliability measures (Goldberg, 1999). Finally, free public domain instruments with high validity enable large-scale assessments without being limited by costly licensing fees (Goldberg et al., 2006).

### Data preparation and analysis

For the 50-item ARSQ (ARSQ 1.0) measurements, data were obtained from a total of 1445 participants, whereas 993 participants completed the recently extended 54-item ARSQ (ARSQ 2.0). In addition to all items included in the ARSQ 1.0, the ARSQ 2.0 contained the following four statements: “I pictured events,” “I pictured places,” “I had silent conversations,” and “I imagined talking to myself” (Table S2). These items served to extend the list of existing ones querying experiences related to images and words, which are important facets of mind wandering (Schooler and Schreiber, 2004; Buckner and Carroll, 2007; Heavey and Hurlburt, 2008; Delamillieure et al., 2010), enabling the specification of the two additional factors “Visual Thought” and “Verbal Thought.” Finally, motivated by applicability in clinical settings, we specified a third factor “Health concern” based on the existing ARSQ statements “I felt pain,” “I felt ill,” and “I thought about my health.” As such, “Health concern” aims to capture variability related to extreme discomfort and preoccupation with health status, often observed in clinical samples (Moffic and Pyakel, 1975).

To keep results compatible with previous data (Diaz et al., 2013), both data sets were filtered based on (1) reported interruption, (2) low motivation (rating “Disagree” or lower), (3) low rated ability to remember thoughts/feelings (rating “Disagree” or lower), (4) reported rating inability (rating below “Agree”), (5) not having eyes closed (rating below “Agree”), and (6) exhibiting extreme responses on the majority of items. This conservative procedure left 882 participants for the ARSQ 1.0 and 562 for the ARSQ 2.0 data set. Data were analyzed using MATLAB 2013a (The Mathworks Inc., Natick, MA), and confirmatory factor analyses (diagonally weighted least squares estimator, unit variance identification, zero mean for the latent variables and unit loading identification) were performed using the Lavaan package (Rosseel, 2012) of R (R Core Team, 2013, Vienna, Austria). Correction for multiple comparisons, where applicable, was performed using false discovery rate (Benjamini and Hochberg, 1995).

## Results

### An improved 10-factor amsterdam resting-state questionnaire

**Figure 1:**
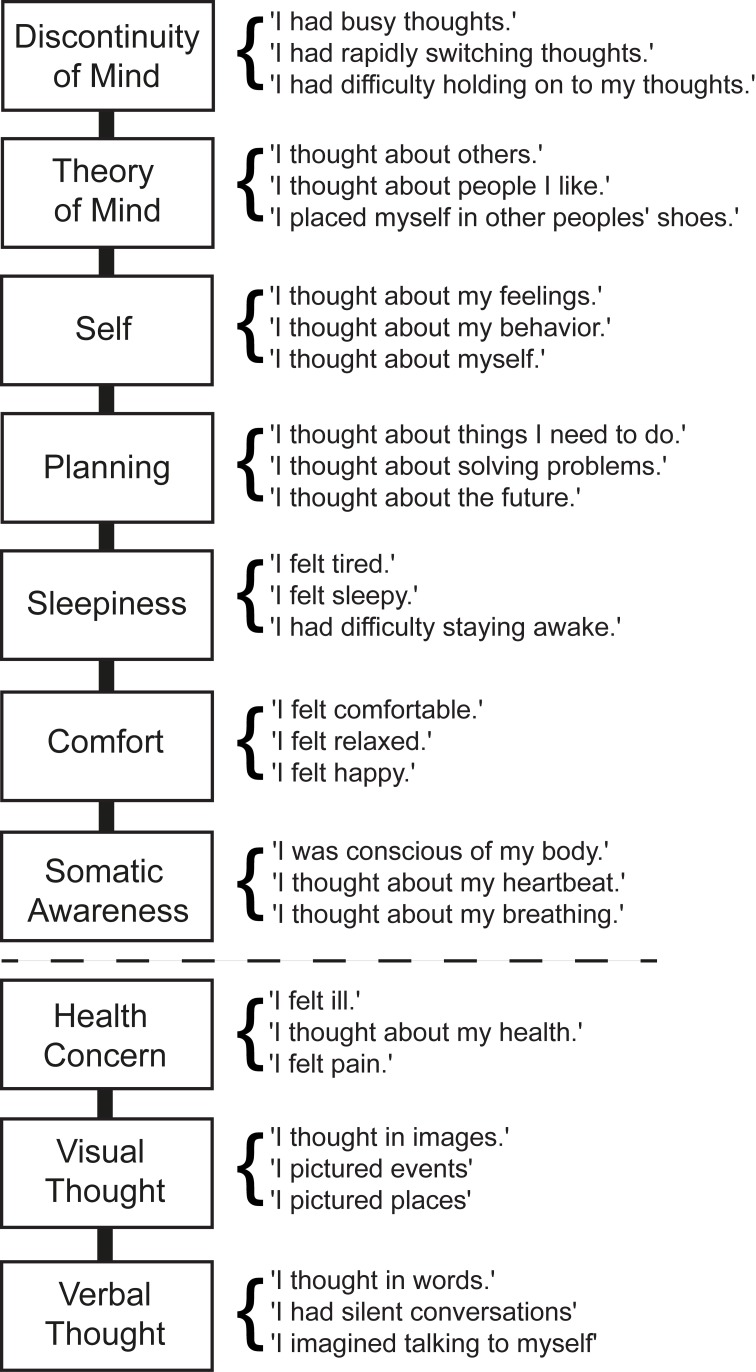
**Ten-factor model of mind wandering derived from the Amsterdam Resting-State Questionnaire.** The existing model of ARSQ-derived mind-wandering factors (above dashed line, Diaz et al., 2013) can be simplified by reducing the number of items per factor to three. Newly added dimensions tap into verbal and visual thought and “Health Concern.”

The dimensional structure of the previously published ARSQ 1.0 (Diaz et al., 2013) harbored an unequal number of items per dimension, thereby introducing a difference in scale-interval between some of the factors. In our attempt to improve upon the efficiency and practicality of the existing ARSQ, we first gathered data from the original ARSQ 1.0 (Table S3) and tested whether a model with a reduced number of items would improve its fit statistics. To this end, the lowest-loading items within each factor were removed, keeping three indicators per factor. The remaining 21 ARSQ items (see Figure 1, above dashed line) were then used to specify a similar seven-factor model of resting-state cognition compared to our earlier report (Diaz et al., 2013). This new model fit the data well: χ^2^_(168, N = 882)_ = 989.71, Root-mean-square error of approximation (RMSEA) = 0.074, Comparative Fit Index (CFI) = 0.97. To show that this fit was not dependent on a specific composition of participants, the same confirmatory factor analysis (CFA) was repeated by selecting 600 random cases from the total set (~67% of the data) 1000 times, yielding satisfactory 95% confidence intervals for CFI [0.96, 0.98] and for RMSEA [0.068, 0.081].

Having shown that the abbreviated 7-factor model fits the ARSQ data well, we then proceeded to test an extended 10-factor model (Figure 1). The newly specified factors were “Health Concern,” “Visual Thought,” and “Verbal Thought,” each with three items, resulting in 30 items in the model. The “Health Concern” factor includes items already present in the original ARSQ 1.0, whereas the factors “Visual Thought” and “Verbal Thought” demanded the addition of 4 new items (Figure 1), resulting in the ARSQ 2.0. This 10-factor model showed acceptable fit [χ^2^_(360, N = 562)_ = 1887.1, *RMSEA* = 0.087, *CFI* = 0.92] and to show that sample composition was unlikely to have affected the results, the confirmatory factor analysis was replicated 1000 times, each time drawing 420 cases at random (75% of the data, to maintain robust results given the smaller sample size). The resulting 95% confidence intervals were satisfactory for both CFI [0.91, 0.93] and RMSEA [0.079, 0.089].

**Figure 2:**
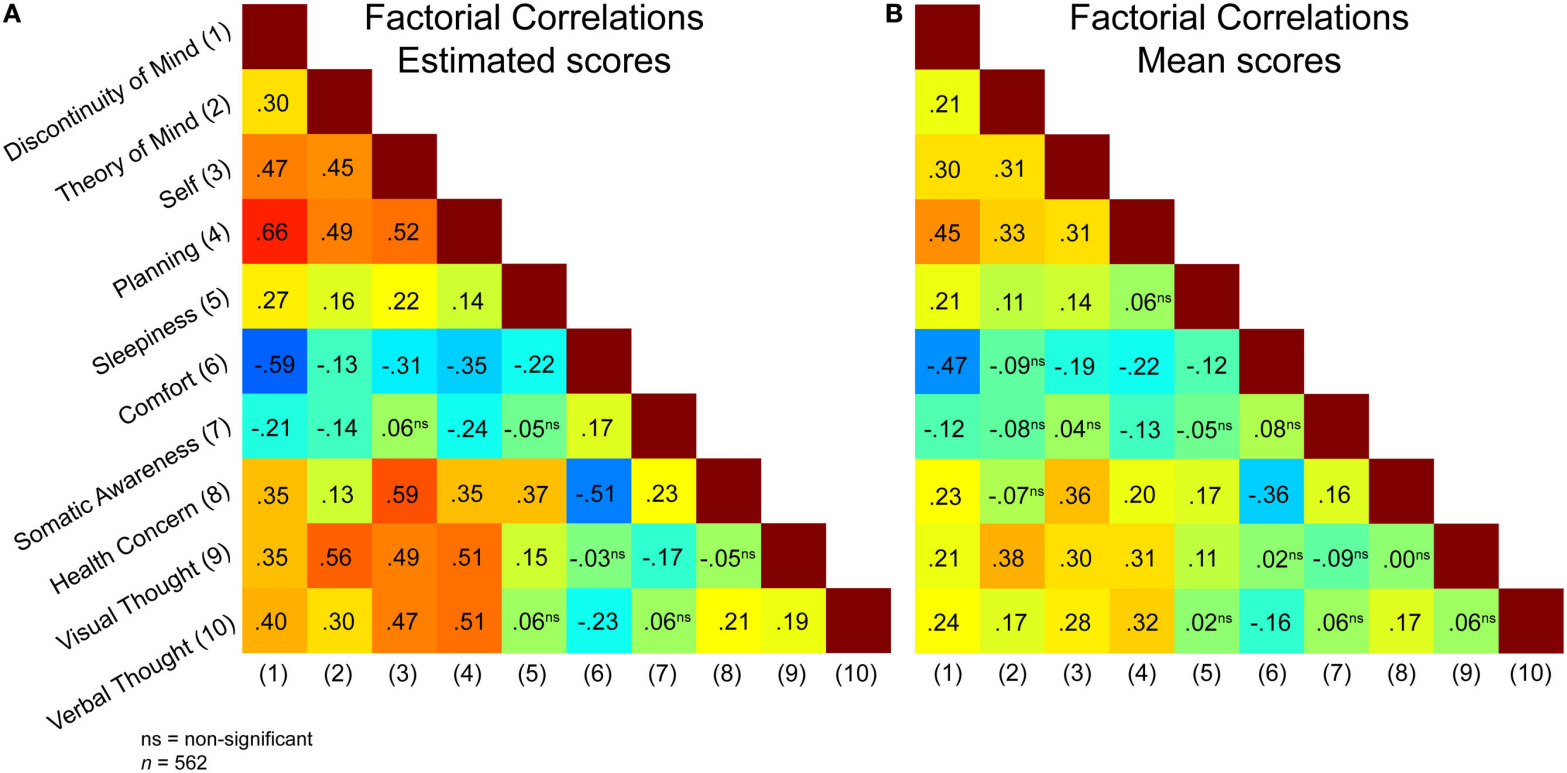
**Relationships among ARSQ dimensions for the 10-factor model.** Factorial correlations among factors for the CFA model **(A)** are similar in magnitude and direction to the correlations among the factors based on mean scores alone **(B)**, supporting the choice for the simplified mean-score approach to quantifying mind wandering.

**Figure 3:**
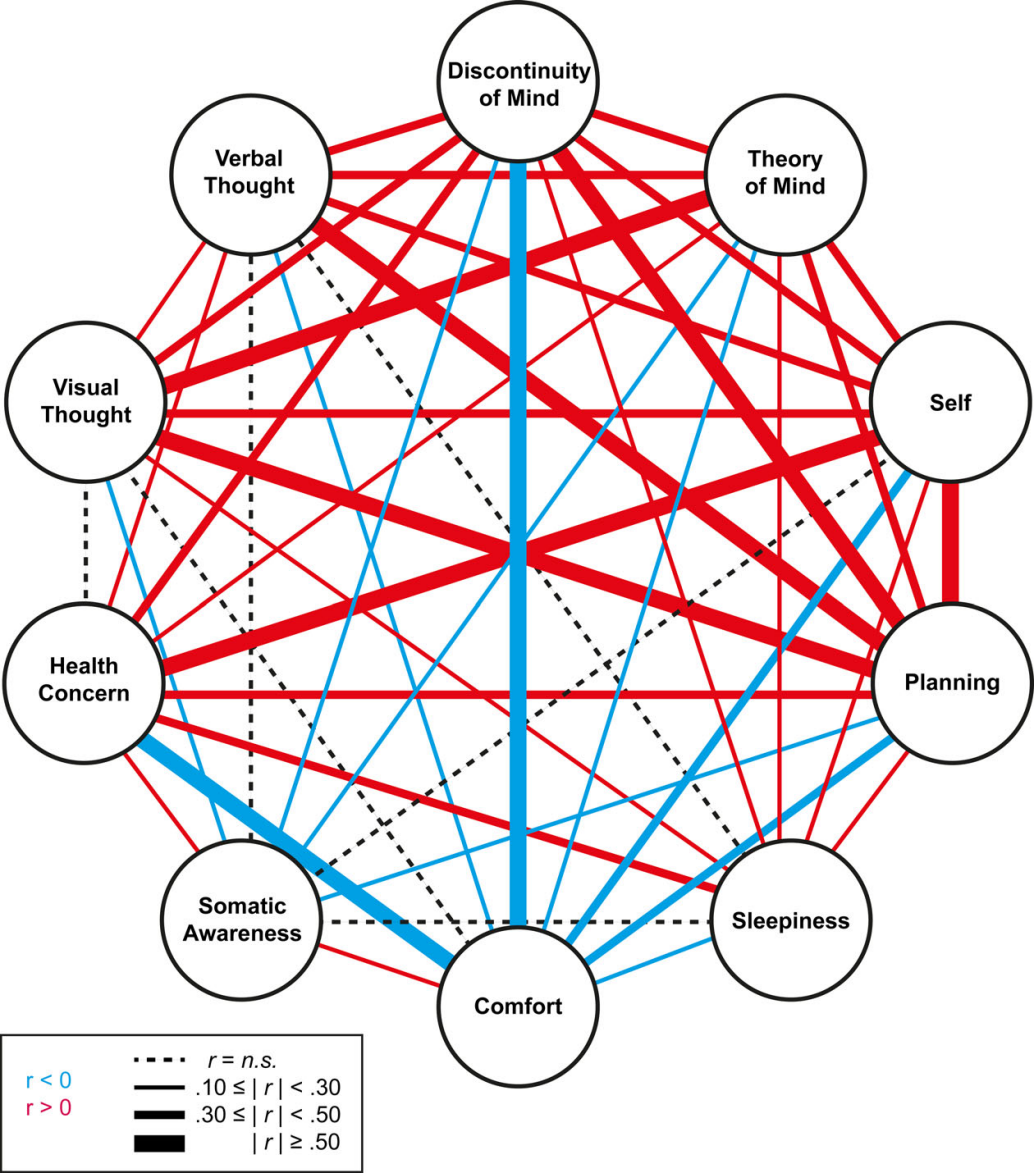
**The intricate web of associations between the 10 dimensions of mind wandering.** To facilitate inspection of the many significant positive (*red*) or negative (*blue*) model-estimated factorial correlations among the 10 dimensions of mind wandering (Figure 2A), these have been shown as pair-wise connections with the line thickness indicating the strength of the effect.

After having tested the newly specified model, we computed scores for each factor based on the mean of the raw ARSQ 2.0 responses of the respective factor items. This procedure favors straightforward applications, as it avoids estimation of individual factor scores per new sample. The averaging of the scores furthermore produces scores on the same [1, 5] interval as the original ARSQ 1.0 statements, aiding interpretation of absolute values. Correlating the estimated ARSQ 2.0 factor scores to the mean scores (Table 1) suggests that compared to our original 7-factor model, reduction of the number of items per factor results in a very good approximation of the estimated factor scores by the mean scores. Notably, the factor “Somatic Awareness” benefits the most from the removal of relatively low-loading items. While using mean scores (as opposed to factor scores) results in lowered factorial correlations, Figure 2B shows that these remain significant and in the same direction as the estimated factorial correlations from the CFA model. The strength and significance of the estimated correlations among the factors is further visualized in Figure 3.

**Table 1:** **Correlation between estimated CFA factor scores and scores derived from averaging the item responses within each factor**.

	**ARSQ 1.0 Correlations 7-factor model^a^ n = 813, *p_corrected_* < 0.001**	**ARSQ 2.0 Correlations 10-factor model n = 562, *p_corrected_* < 0.001**
Discontinuity of mind	0.95	0.91
Theory of mind	0.98	0.97
Self	0.88	0.90
Planning	0.96	0.90
Sleepiness	0.96	0.96
Comfort	0.94	0.91
Somatic awareness	0.66	0.94
Health concern	–	0.90
Visual thought	–	0.87
Verbal thought	–	0.92

The Pearson correlation coefficients indicate that the mean scores are good predictors of the estimated factor scores also in the ARSQ 2.0 based 10-factor model.

a From (Diaz et al., 2013), p-values false discovery rate corrected.

Finally, to account for potential age and gender effects, we tested the effect of the fixed factor gender on the 10 mean scores (*n* = 562) using multivariate ANOVA, taking age as covariate. The results indicated no overall effect of gender [*F*_(10, 541)_ = 1.61, *p* = 0.10] on mind wandering. The only significant difference in mean scores between the genders was found for “Verbal Thought” [*F*_(10, 550)_ = 4.23, *p* = 0.04], with women indicating to have experienced slightly more verbal thoughts (*M* = 3.1, *SD* = 0.86) compared to men (*M* = 2.85, *SD* = 0.86).

However, there was a significant effect of the covariate age [*F*_(10, 541)_ = 6.51, *p* < 0.001]. A subsequent correlation analysis between age and the mean scores of the 10-factors showed significant negative correlations for both “Discontinuity of Mind” [*r*_(560)_ = -0.20, *p* < 0.001], “Self,” [*r*_(560)_ = −0.18, *p* < 0.001], “Planning,” [*r*_(560)_ = −0.27, *p* < 0.001], “Visual Thought,” [*r*_(560)_ = −0.20, *p* < 0.001], and “Verbal Thought,” [*r*_(560)_ = −0.15, *p* < 0.001]. These results suggest that people experience less verbal and visual thought, less planning and fewer thoughts about themselves with increasing age. To investigate this further, we tested for an age effect on the single items “I felt nothing” and “I thought about nothing,” which are part of the full ARSQ 1.0 and ARSQ 2.0 albeit not part of any factor solution. Interestingly, we indeed identified a small yet significant increase in the response to these items with age [both *r*_(560)_ = 0.16, *p* < 0.001].

### Stability of ARSQ factors over time

Recent reports have shown that ARSQ scores are quite stable over a short interval of 45 min (Diaz et al., 2013). This raises the question to what extent ARSQ responses are stable over longer durations. A subset of participants (*n* = 216) filled out both the ARSQ 1.0 and ARSQ 2.0 with a median interval between both measurements of 16.6 months (range: 2.9–31.5 months). We tested the equality of test-retest correlations over time by dividing the total sample (*n* = 216) into 3 equal subgroups (G1: *n* = 72, time between administrations 2.9–14.2 months; G2: *n* = 72, time between administrations 14.3–24.6 months; G3: *n* = 72, time between administrations 24.7–31.5 months). Because the original ARSQ 1.0 lacked the items to form the factors “Visual Thought” and “Verbal Thought,” only the single items “I thought in images” and “I thought in words” were used as predictor variables. We observed test-retest correlations of mean scores ranging between 0.34 and 0.54 for the 10 dimensions of the ARSQ (Table 2). This suggests that there is a stable component to mind wandering, except for “Sleepiness” and “Health Concern,” which are indeed expected to fluctuate on time scales of days or months as part of the normal variation in the amount of sleep obtained or health status.

**Table 2:** **Test-retest correlations between first (ARSQ 1.0) and second (ARSQ 2.0) assessments based on mean scores**.

	***r_G1_***	***r_G2_***	***r_G3_***	***r_averaged_***
Discontinuity of mind	0.55	0.50	0.56	0.54
Theory of mind	0.31	0.33	0.29	0.31
Self	0.44	0.41	0.45	0.43
Planning	0.36	0.13	0.28	0.26
Sleepiness	0.12	0.30	0.57	0.34^*^
Comfort	0.36	0.56	0.49	0.47
Somatic awareness	0.55	0.28	0.45	0.43
Health concern	0.67	0.45	0.26	0.47^*^
Visual thought^1^	0.37	0.23	0.31	0.31
Verbal thought^2^	0.35	0.12	0.35	0.27

Testing for equal correlations over three time periods (G1: 2.9–14.2 months; G2: 14.3–24.6 months; G3: 24.7–31.5 months, n = 72 for each group) revealed significant differences only for “Sleepiness” and “Health Concern” (asterisks). This suggests that all other shown factors were stable across the three time intervals as indicated by their averaged correlation.

*n = 216,* ^1^*Single item “I thought in images,”* ^2^*Single item “I thought in words,”*

* Correlation showing significant (false discovery rate corrected) differences over groups.

### Mind wandering and personality traits

To address the question whether the stability in ARSQ factors is related to personality, mean scores for the ARSQ 2.0 sample were correlated with the 3 main character and 4 main temperament dimensions obtained from the personality inventory we administered (see Materials and Methods). As predicted by the psychobiological model (Cloninger et al., 1993; De Fruyt et al., 2000), the character dimensions—which are more dependent on conscious, episodic memory-driven experiences—appeared to be correlated to a larger set of ARSQ factors than the temperaments, which are theorized to reflect more unconscious tendencies (Table 3). Notably, “Self-Directedness,” an indicator of how well an individual can adapt, control and regulate behavior in response to situational changes and in line with personal goals, appears significantly correlated with most ARSQ factors. For instance, more self-directedness is associated with more control over thoughts (i.e., lower scores on “Discontinuity of Mind”) and higher scores on Comfort. By contrast, “Self-Transcendence,” associated with spiritual traits (e.g., engaging in prayer or meditation), appeared to be largely independent of ARSQ factors. Contrary to expectation, the trait “Cooperativeness,” which should reflect individual differences in identification with, and acceptance of others (Cloninger et al., 1993), showed only few significant (and small) correlations with ARSQ factors and did not correlate with “Theory of Mind.” On the other hand, “Theory of Mind” was significantly related to “Reward Dependence,” viewed as a heritable bias toward seeking approval of and attachment to others (Cloninger et al., 1993). Other temperaments associated with ARSQ factors include “Harm Avoidance,” which has been related to neuroticism and fear of uncertainty (De Fruyt et al., 2006) and showed a negative correlation with Comfort and a positive correlation with Health Concern. By contrast, “Novelty Seeking,” assumed to reflect impulsivity, showed a positive correlation with Comfort and a negative correlation with Health Concern. Taken together, several of the significant correlations appeared plausible in direction albeit small in magnitude.

**Table 3:** **Correlation matrix of ARSQ 2.0 factors and IPIP personality dimensions based on Cloninger's psychobiological model (Cloninger et al., 1993; Goldberg et al., 2006)**.

	**Self-directedness**	**Cooperativeness**	**Self-transcendence**	**Novelty seeking**	**Harm avoidance**	**Reward dependence**	**Persistence**
Discontinuity of mind	−0.26^*^	−0.16^*^	−0.11^*^	−0.17^*^	0.16^*^	0.03	−0.09
Theory of mind	−0.06	0.03	−0.04	−0.02	0.04	0.15^*^	−0.01
Self	−0.20^*^	−0.11^*^	−0.02	0.01	0.04	0.11^*^	−0.12^*^
Planning	−0.18^*^	−0.16^*^	−0.07	−0.04	0.07	−0.01	0.00
Sleepiness	−0.12^*^	−0.06	0.01	−0.05	0.04	0.03	−0.09
Comfort	0.30^*^	0.13^*^	0.07	0.22^*^	−0.32^*^	0.02	0.20^*^
Somatic awareness	0.03	0.08	0.06	0.05	−0.01	0.09	0.02
Health concern	−0.18^*^	0.01	0.01	−0.13^*^	0.20^*^	0.02	−0.07
Visual thought	−0.08	−0.12^*^	−0.08	0.01	0.01	0.03	−0.01
Verbal thought	−0.12^*^	0.01	0.01	−0.01	0.05	0.07	−0.04

* *p < 0.01, all p-values corrected using false discovery rate, n = 502*.

To test for age and gender effects on the character and temperament traits, multivariate ANOVA was performed, with gender as fixed-factor and age as covariate. Both the overall main effect of gender [*F*_(7, 492)_ = 8.61, *p* = 0.001] and the covariate age [*F*_(7, 492)_ = 6.45, *p* = 0.001] were significant. Closer inspection of the gender effect revealed higher average “Self-Transcendence” [*F*_(1, 498)_ = 14.23, *p* = 0.001], “Harm Avoidance” [*F*_(1, 498)_ = 6.81, *p* = 0.01], and “Reward Dependence” [*F*_(1, 498)_ = 14.18, *p* = 0.001] for women [*M* = (7.02, 7.78, 12.95), *SD* = (1.58, 2.92, 2.79)] compared to men [*M* = (6.52, 7.00, 11.81), *SD* = (1.61, 3.16, 3.05)], respectively. Finally, a correlation analysis revealed that age had a significant, but low association with self-directedness [*r*_(500)_ =0.11, *p* < 0.01], cooperativeness [*r*_(500)_ = 0.15, *p* < 0.01], self-transcendence [*r*_(500)_ = 0.14, *p* < 0.01], and novelty-seeking [*r*_(500)_ = 0.12, *p* < 0.01]. These small correlations are in line with the expectation that personality traits change little with age (McCrae and Costa, 1994).

## Discussion

The past decade has witnessed a markedly increasing interest in resting-state brain activity, mind wandering and their putative associations (Smallwood and Schooler, 2006; Buckner and Carroll, 2007; Raichle, 2011; Fell, 2013). The resting-state condition also features prominently in clinical research, because of the ease with which patients can perform the task (Linkenkaer-Hansen et al., 2005; Andrews-Hanna et al., 2007; Stoffers et al., 2007; Greicius, 2008; Montez et al., 2009). In spite of the progress that has been achieved on the neuroimaging side, however, efforts toward efficient assessment and quantification of the subjective dimension of mind wandering have been limited (Delamillieure et al., 2010). In our view, integration of standard neuroimaging methodology and the assessment of subjective experience is necessary in order to gain a more complete understanding of mind wandering in general and the functional significance of resting-state brain activity in particular. This formed the driving force behind the development of the ARSQ, a time-efficient and informative tool for resting-state and mind-wandering research (Diaz et al., 2013).

The current study builds on our earlier efforts, yielding a model of mind wandering with more factors, acknowledging the earlier underrepresentation of important factors such as imagery and inner speech (Schooler and Schreiber, 2004; Heavey and Hurlburt, 2008; Delamillieure et al., 2010) and a standardized set of items per factor in order to avoid differences in the discreetness of the underlying scale. This updated model performs well on a theoretical basis (i.e., improved fit statistics for CFI) and is well approximated by mean item scores. Finally, the addition of a separate factor for “Health Concern,” provides an extra estimator of the experience or concern of being ill, often associated with for example patients suffering from depression or chronic pain (Moffic and Pyakel, 1975; Sarnthein et al., 2006).

Our previous results have shown that ARSQ mean scores retain high test-retest correlations over 45 min. Interestingly, even at (much) longer time scales spanning several months between assessments, most correlations remain significant, albeit weaker. Only the factors “Sleepiness” and “Health Concern” did not exhibit stability over time, which is understandable as health status can naturally change over time and sleepiness may be a highly dynamic state (i.e., affected by a restless night, time of day, etc.). To further identify potential contributors to the observed stability in mind wandering, we correlated the ARSQ mean scores to personality measures. We obtained scores on a subset of items closely related to Cloninger's Temperament and Character Inventory (Cloninger et al., 1993; De Fruyt et al., 2000; Goldberg et al., 2006), measuring three characters, closely related to conscious concept-driven processing and four temperaments, measuring largely unconscious behavioral tendencies. Considering the prominent role of conscious experience in both mind wandering and Cloninger's definition of character traits, we expected significant associations between the ARSQ factors and the three character dimensions. Our results supported this hypothesis partially. The character trait Self-Directedness correlated significantly with most ARSQ factors, and interestingly all significant correlations except for the relation with “Comfort,” proved negative. This may suggest that a higher disposition on self-directedness, defined as the individual ability to govern behavior according to situational demands and in line with personal motivators (Cloninger et al., 1993; Watson and Tharp, 2014), is related to *less* mind wandering and *higher* ratings of comfort. Still, the other character traits exhibited far fewer correlations with the ARSQ factors. For example, the character trait Cooperativeness did not correlate with “Theory of Mind,” which appears counter-intuitive, as this trait theoretically measures the individual ability to identify with and accept other people—descriptions that fit well with the concept of theory of mind. The temperaments on the other hand, showed specific correlations with up to three ARSQ factors only (Table 3). The ARSQ factor “Comfort” appears to be most readily associated with temperaments, adding to face validity via a negative correlation with the neuroticism-related trait Harm Avoidance and a positive relation with Persistence, measuring self-confidence and perseverance.

Overall, our results suggest that the traits of the psychobiological model explain little in terms of variability in ARSQ factors. The possibility exists that other personality inventories, such as the well-known NEO-PI-R (Costa and McCrae, 1985), which measures the “Big Five” (i.e., neuroticism, extraversion, conscientiousness, agreeability and intellect) would reveal stronger correlations. Still, previous studies have revealed relatively strong correlations between the Big Five and the dimensions of Cloninger's Temperament and Character inventory (De Fruyt et al., 2006). The search to disentangle stable trait components from more dynamic states and their interaction is an active field of research. Rigorous tracking of mind wandering over longer periods, comparable to “experience sampling” (Zelenski and Larsen, 2000), in combination with advanced statistical methodology such as CFA/structural equation modeling (Steyer et al., 1999) and novel theoretical frameworks, such as conceptualizing traits as state density functions (Fleeson, 2001), may yield a more detailed picture of the state-trait interactions in relation to mind wandering. Current findings suggest that at least part of the ARSQ retest correlations may be governed by “trait-like” components, possibly genetic in nature, and mind-wandering research may therefore benefit from these novel approaches. Alternatively, the (subset of) TCI items we have utilized in this study may be too heterogeneous, i.e., measuring too many distinct facets, preventing clear-cut correlations with the ARSQ. However, inspection of the individual items used (Table S1) for the character “Cooperativeness” and temperament “Reward Dependence” strongly suggests they aim to capture an individual's disposition towards other people, hence they appear congruent with the “Theory of Mind” items of the ARSQ. Perhaps then, there is a discrepancy between how people generally rate themselves, e.g., in personality inventories and their actual experiences during mind wandering, which may be heavily self-referential as suggested by the high factorial correlations for the ARSQ factor “Self” (Figure 2).

Finally, we investigated whether age and gender were associated with mind-wandering experience and personality traits. Although both gender and age effects on personality were in line with earlier reports (McCrae and Costa, 1994), only age appeared to have a significant main negative effect on ARSQ dimensions “Planning,” “Self,” and “Visual Thought.” These findings are supported by earlier reports of diminished “current concerns” with age (Klinger, 1999; McVay and Kane, 2010) and could allow future studies to confirm whether age-related variation in neuroimaging measurements can be related to mind wandering.

To conclude, the here presented extended ARSQ and associated 10-factor model improves on the original ARSQ by introducing important additional dimensions. We propose to refer to this revision as “ARSQ 2.0”^1^ to distinguish it from the previous version (Diaz et al., 2013). We remain confident that a tool such as the ARSQ will help further develop the “neuroscience of mind wandering” (Gruberger et al., 2011) and help bridge the gap between its behavioral and subjective dimensions.

## Conflict of interest statement

The authors declare that the research was conducted in the absence of any commercial or financial relationships that could be construed as a potential conflict of interest.

## Footnotes

^1^The ARSQ is available in multiple translations on request.
