# Supplementary material for: The ARSQ 2.0 reveals age and personality effects on mind-wandering experiences
Source: Front Psychol. 2014 Apr 3;5:271. doi: 10.3389/fpsyg.2014.00271 (PMC3982068; doi:10.3389/fpsyg.2014.00271)
Supplement: Supplementary file 1 [file DataSheet1.DOCX]

**Supplementary Material**

**The extended Amsterdam Resting-State Questionnaire—relation to age, gender and personality**

B. Alexander Diaz^a,e^, Sophie Van Der Sluis^b,e^, Jeroen S. Benjamins^c^, Diederick Stoffers^c^, Richard Hardstone^a,e^, Huibert D. Mansvelder^a,e^, Eus J.W. Van Someren^a,c,d,e^, and Klaus Linkenkaer-Hansen^a,e*^

^a^Department of Integrative Neurophysiology, Center for Neurogenomics and Cognitive Research (CNCR), VU University Amsterdam, De Boelelaan 1085, 1081 HV Amsterdam, The Netherlands.

^b^Department of Functional Genomics, Center for Neurogenomics and Cognitive Research (CNCR), VU University Amsterdam & VU Medical Center Amsterdam, De Boelelaan 1085, 1081 HV Amsterdam, The Netherlands.

^c^Department of Sleep & Cognition, Netherlands Institute for Neuroscience, Meibergdreef 47, 1105 BA Amsterdam, The Netherlands.

^d^Department of Medical Psychology, VU University Medical Center, 1Y 156, De Boelelaan 1117, 1081 HZ Amsterdam

^e^Neuroscience Campus Amsterdam (NCA), De Boelelaan 1085, 1081 HV Amsterdam, The Netherlands.

**Table S1**. List of IPIP personality items (Goldberg et al., 2006) used in the current study (R denotes reverse coding prior to analysis), which are similar in nature to Cloninger’s Temperament and Character Inventory (Cloninger et al., 1993; De Fruyt et al., 2000).

| **Item** | **Dimension** | **Sub-dimension** | **Coding** |
| --- | --- | --- | --- |
| Am quick to judge others. | *Cooperativeness* | C1 Tolerance | R |
| Am a bad loser. |  | C1 Tolerance | R |
| Treat people as inferiors. |  | C1 Tolerance | R |
| Accept people as they are. |  | C1 Tolerance |  |
| Believe that others have good intentions. |  | C1 Tolerance |  |
| Respect others. |  | C1 Tolerance |  |
| Make others feel good. |  | C2 Empathy |  |
| Make people feel welcome. |  | C2 Empathy |  |
| Reassure others. |  | C2 Empathy |  |
| Take time out for others. |  | C2 Empathy |  |
| Can't be bothered with other's needs. |  | C3 Trust | R |
| Disregard the opinions of others. |  | C3 Trust | R |
| Distrust people. |  | C3 Trust | R |
| Am nice to store clerks. |  | C3 Trust |  |
| Trust others. |  | C3 Trust |  |
| Try to maintain a pleasant atmosphere. |  | C3 Trust |  |
| Find it hard to forgive others. |  | C4 Compassion | R |
| Get angry easily. |  | C4 Compassion | R |
| Get back at others. |  | C4 Compassion | R |
| Look down on others. |  | C4 Compassion | R |
| Accept others' weaknesses. |  | C4 Compassion |  |
| Am inclined to forgive others. |  | C4 Compassion |  |
| Try to forgive and forget. |  | C4 Compassion |  |
| Believe that the end justifies the means. |  | C5 Morality | R |
| Misuse power. |  | C5 Morality | R |
| Try to fool others. |  | C5 Morality | R |
| Care about justice. |  | C5 Morality |  |
| Listen to my conscience. |  | C5 Morality |  |
| Return extra change when a cashier makes a mistake. |  | C5 Morality |  |
| Fear for the worst. | *Harm Avoidance* | HA1 Neuroticism |  |
| Enjoy being reckless. |  | HA2 Harm Avoidance | R |
| Take risks. |  | HA2 Harm Avoidance | R |
| Avoid dangerous situations. |  | HA2 Harm Avoidance |  |
| Would never go hang gliding or bungee jumping. |  | HA2 Harm Avoidance |  |
| Would never make a high risk investment. |  | HA2 Harm Avoidance |  |
| Am comfortable in unfamiliar situations. |  | HA3 Social Discomfort | R |
| Am not bothered by difficult social situations. |  | HA3 Social Discomfort | R |
| Am quiet around strangers. |  | HA3 Social Discomfort |  |
| Find it difficult to approach others. |  | HA3 Social Discomfort |  |
| Only feel comfortable with friends. |  | HA3 Social Discomfort |  |
| Can manage many things at the same time. |  | HA4 Low Self-efficacy | R |
| Can tackle anything. |  | HA4 Low Self-efficacy | R |
| Am afraid of many things. |  | HA4 Low Self-efficacy |  |
| Feel that I'm unable to deal with things. |  | HA4 Low Self-efficacy |  |
| Dislike changes. | *Novelty Seeking* | NS1 Variety Seeking | R |
| Don't like the idea of change. |  | NS1 Variety Seeking | R |
| Prefer to stick with things that I know. |  | NS1 Variety Seeking | R |
| Am open to change. |  | NS1 Variety Seeking |  |
| Like to begin new things. |  | NS1 Variety Seeking |  |
| Prefer variety to routine. |  | NS1 Variety Seeking |  |
| Have an eye for detail. |  | NS2 Recklessness | R |
| Like to sleep on things before acting. |  | NS2 Recklessness | R |
| Think twice before doing something. |  | NS2 Recklessness | R |
| Like to act on a whim. |  | NS2 Recklessness |  |
| Make rash decisions. |  | NS2 Recklessness |  |
| Never abuse my credit. |  | NS3 Extravagance | R |
| Never spend more than I can afford. |  | NS3 Extravagance | R |
| Overuse my credit. |  | NS3 Extravagance |  |
| Spend more money than I have. |  | NS3 Extravagance |  |
| Respect authority. |  | NS4 Rebelliousness | R |
| Would never cheat on my taxes. |  | NS4 Rebelliousness | R |
| Break rules. |  | NS4 Rebelliousness |  |
| Resist authority. |  | NS4 Rebelliousness |  |
| Find it difficult to get down to work. | *Persistence* | P1 Initiative | R |
| Have difficulty starting tasks. |  | P1 Initiative | R |
| Put off unpleasant tasks. |  | P1 Initiative | R |
| Finish tasks quickly. |  | P1 Initiative |  |
| Plunge into tasks with all my heart. |  | P1 Initiative |  |
| Don't put my mind on the task at hand. |  | P2 Competence | R |
| Don't see things through. |  | P2 Competence | R |
| Give up easily. |  | P2 Competence | R |
| Feel up to any task. |  | P2 Competence |  |
| Like to solve complex problems. |  | P2 Competence |  |
| Meet challenges. |  | P2 Competence |  |
| Want to be the very best. |  | P3 Achievement-striving |  |
| Do just enough work to get by. |  | P4 Industriousness | R |
| Put little time and effort into my work. |  | P4 Industriousness | R |
| Do more than what's expected of me. |  | P4 Industriousness |  |
| Set high standards for myself and others. |  | P4 Industriousness |  |
| Work hard. |  | P4 Industriousness |  |
| Am indifferent to the feelings of others. | *Reward Dependence* | RD1 Sentimentality | R |
| Don't have a soft side. |  | RD1 Sentimentality | R |
| Seldom get emotional. |  | RD1 Sentimentality | R |
| Cry easily. |  | RD1 Sentimentality |  |
| Am hard to get to know. |  | RD2 Friendliness | R |
| Keep others at a distance. |  | RD2 Friendliness | R |
| Am interested in people. |  | RD2 Friendliness |  |
| Enjoy bringing people together. |  | RD2 Friendliness |  |
| Bottle up my feelings. |  | RD3 Self-disclosure | R |
| Reveal little about myself. |  | RD3 Self-disclosure | R |
| Am open about myself to others. |  | RD3 Self-disclosure |  |
| Show my feelings. |  | RD3 Self-disclosure |  |
| Believe only in myself. |  | RD4 Dependence | R |
| Don't care what others think. |  | RD4 Dependence | R |
| Want to be different from others. |  | RD4 Dependence | R |
| Do what others want me to do. |  | RD4 Dependence |  |
| Hate to seem pushy. |  | RD4 Dependence |  |
| Try to please everyone. |  | RD4 Dependence |  |
| Feel attacked by others. | *Self-directedness* | S1 Satisfaction | R |
| Feel short-changed in life. |  | S1 Satisfaction | R |
| Feel that people have a hard time understanding me. |  | S1 Satisfaction | R |
| Let myself be used. |  | S1 Satisfaction | R |
| Withhold information from others. |  | S1 Satisfaction | R |
| Know how to enjoy myself. |  | S1 Satisfaction |  |
| Am not sure where my life is going. |  | S2 Optimism | R |
| Feel that my life lacks direction. |  | S2 Optimism | R |
| Let others determine my choices. |  | S2 Optimism | R |
| Know what I want. |  | S2 Optimism |  |
| Love life. |  | S2 Optimism |  |
| Am easily discouraged. |  | S3 Resourcefulness | R |
| Am easily intimidated. |  | S3 Resourcefulness | R |
| Panic easily. |  | S3 Resourcefulness | R |
| Am good at many things. |  | S3 Resourcefulness |  |
| Can handle complex problems. |  | S3 Resourcefulness |  |
| Formulate ideas clearly. |  | S3 Resourcefulness |  |
| Am out for my own personal gain. |  | S4 Self-acceptance | R |
| Look down on any weakness. |  | S4 Self-acceptance | R |
| See other people as my competitors. |  | S4 Self-acceptance | R |
| Seek status. |  | S4 Self-acceptance | R |
| Respect the opinions of others. |  | S4 Self-acceptance |  |
| Don't know why I do some of the things I do. |  | S5 Impulse Control | R |
| Make a mess of things. |  | S5 Impulse Control | R |
| Waste my time. |  | S5 Impulse Control | R |
| Easily resist temptations |  | S5 Impulse Control |  |
| Carry out my plans. |  | S5 Impulse Control |  |
| Rarely overindulge. |  | S5 Impulse Control |  |
| Am inexplicably happy some of the time. | *Self-transcendence* | ST2 Romanticism |  |
| Love flowers. |  | ST2 Romanticism |  |
| Believe that there is no absolute right or wrong. |  | ST4 Conservatism | R |
| Dislike movies with happy Hollywood endings. |  | ST4 Conservatism | R |
| Tend to vote for liberal political candidates. |  | ST4 Conservatism | R |
| Believe in the importance of tradition. |  | ST4 Conservatism |  |
| Tend to vote for conservative political candidates. |  | ST4 Conservatism |  |
| Try not to think about the needy. |  | ST5 Femininity | R |
| Feel sympathy for those who are worse off than myself. |  | ST5 Femininity |  |
| Love children. |  | ST5 Femininity |  |
| Radiate joy. |  | ST5 Femininity |  |

**Table S2**. Endorsement rate of the Amsterdam Resting-State Questionnaire 2.0 filtered data set (*n* = 562). Newly added items (N) for the visual and verbal thoughts factors, reverse coded items (R) and validation items (V) indicated as such. Ratings ranged from “Completely disagree” (--) to “Completely agree” (++) on a 5-point rating scale.

| **Item Label** | **--** | **-** | **+/-** | **+** | **++** |
| --- | --- | --- | --- | --- | --- |
| I thought about my feelings. | 7 | 26 | 18 | 44 | 4 |
| I felt restless. | 19 | 41 | 18 | 20 | 3 |
| I felt tired. | 19 | 43 | 17 | 20 | 2 |
| I felt sleepy. | 20 | 43 | 17 | 19 | 1 |
| I felt comfortable. | 2 | 9 | 24 | 53 | 12 |
| I felt relaxed. | 3 | 11 | 22 | 51 | 13 |
| I felt happy. | 2 | 7 | 45 | 38 | 8 |
| I enjoyed the session. | 7 | 20 | 40 | 29 | 4 |
| I felt bored. | 24 | 40 | 16 | 16 | 3 |
| I felt nothing. | 31 | 45 | 18 | 5 | 1 |
| I felt the same throughout the session. | 0 | 18 | 9 | 63 | 9 |
| I thought about my health. | 22 | 44 | 15 | 16 | 3 |
| I thought about my work/study. | 26 | 32 | 10 | 26 | 6 |
| I thought about my behavior. | 18 | 42 | 23 | 17 | 1 |
| I had thoughts that I would not readily share with others. | 34 | 47 | 9 | 8 | 2 |
| I had busy thoughts. | 12 | 32 | 20 | 28 | 8 |
| I had similar thoughts throughout the session. | 6 | 34 | 18 | 38 | 4 |
| I thought about others. | 10 | 21 | 11 | 51 | 7 |
| I thought about myself. | 6 | 17 | 25 | 46 | 6 |
| I thought about pleasant things. | 4 | 15 | 38 | 38 | 6 |
| I thought about solving problems. | 19 | 32 | 14 | 30 | 4 |
| I thought about the aim of the experiment. | 23 | 34 | 13 | 26 | 3 |
| I had difficulty staying awake. | 41 | 40 | 11 | 6 | 2 |
| I had rapidly switching thoughts. | 5 | 22 | 20 | 40 | 13 |
| I had superficial thoughts. | 6 | 22 | 28 | 41 | 3 |
| I thought about the past. | 22 | 38 | 16 | 22 | 2 |
| I thought about the present. | 1 | 8 | 12 | 69 | 9 |
| I thought about the future. | 16 | 20 | 18 | 38 | 8 |
| I had deep thoughts. | 19 | 39 | 23 | 15 | 3 |
| I thought about nothing. | 41 | 37 | 12 | 8 | 2 |
| I had difficulty holding on to my thoughts. | 7 | 38 | 25 | 25 | 5 |
| I thought about people I like. | 13 | 20 | 20 | 39 | 8 |
| I thought in images. | 8 | 28 | 18 | 37 | 9 |
| I thought in words. | 6 | 20 | 17 | 46 | 11 |
| I thought about things I need to do. | 10 | 24 | 14 | 40 | 11 |
| I was conscious of my body. | 4 | 9 | 13 | 52 | 23 |
| I thought about the sounds around me. | 11 | 29 | 21 | 34 | 6 |
| I thought about the odors around me. | 38 | 48 | 9 | 4 | 0 |
| I thought about my heartbeat. | 41 | 31 | 9 | 17 | 3 |
| I thought about my breathing. | 17 | 26 | 11 | 31 | 15 |
| I placed myself in other people’s shoes. | 23 | 37 | 22 | 17 | 2 |
| I had negative feelings. | 34 | 34 | 17 | 12 | 2 |
| I had my thoughts under control. (R) | 3 | 20 | 32 | 40 | 5 |
| I felt ill. | 61 | 33 | 5 | 1 | 0 |
| I felt pain. | 45 | 37 | 7 | 10 | 1 |
| I pictured events. (N) | 8 | 26 | 18 | 43 | 7 |
| I pictured places. (N) | 17 | 38 | 15 | 26 | 4 |
| I had silent conversations. (N) | 9 | 31 | 14 | 39 | 6 |
| I imagined talking to myself | 14 | 35 | 14 | 33 | 4 |
| I felt motivated to participate. (V) | 0 | 0 | 23 | 60 | 18 |
| I have difficulty remembering my thoughts. (V) | 33 | 54 | 13 | 0 | 0 |
| I have difficulty remembering my feelings. (V) | 19 | 63 | 18 | 0 | 0 |
| I had my eyes closed. (V) | 0 | 0 | 0 | 43 | 57 |
| I was able to rate the statements. (V) | 0 | 0 | 0 | 64 | 36 |

**Table S3**. Endorsement rate of the Amsterdam Resting-State Questionnaire 1.0 as described in (Diaz et al., 2013) filtered data set (*n* = 882). Reverse coded items (R) and validation items (V) indicated as such. Ratings ranged from “Completely disagree” (--) to “Completely agree” (++) on a 5-point rating scale.

| **Item Label** | **--** | **-** | **+/-** | **+** | **++** |
| --- | --- | --- | --- | --- | --- |
| I thought about my feelings. | 22 | 27 | 20 | 26 | 4 |
| I felt restless. | 35 | 28 | 19 | 13 | 5 |
| I felt tired. | 31 | 26 | 21 | 16 | 5 |
| I felt sleepy. | 26 | 32 | 19 | 18 | 4 |
| I felt comfortable. | 1 | 9 | 17 | 57 | 16 |
| I felt relaxed. | 2 | 12 | 19 | 50 | 17 |
| I felt happy. | 4 | 13 | 42 | 33 | 8 |
| I enjoyed the session. | 10 | 20 | 38 | 25 | 7 |
| I felt bored. | 29 | 33 | 21 | 15 | 3 |
| I felt nothing. | 30 | 35 | 25 | 8 | 1 |
| I felt the same throughout the session. | 4 | 19 | 15 | 50 | 13 |
| I thought about my health. | 39 | 31 | 12 | 16 | 2 |
| I thought about my work/study. | 35 | 24 | 10 | 25 | 5 |
| I thought about my behavior. | 33 | 32 | 13 | 20 | 2 |
| I had thoughts that I would not readily share with others. | 54 | 31 | 7 | 6 | 2 |
| I had busy thoughts. | 29 | 32 | 19 | 14 | 6 |
| I had similar thoughts throughout the session. | 6 | 19 | 21 | 44 | 10 |
| I thought about others. | 17 | 24 | 19 | 35 | 5 |
| I thought about myself. | 7 | 12 | 18 | 54 | 9 |
| I thought about pleasant things. | 8 | 17 | 36 | 34 | 6 |
| I thought about solving problems. | 25 | 31 | 16 | 26 | 2 |
| I thought about the aim of the experiment. | 29 | 28 | 15 | 24 | 5 |
| I had difficulty staying awake. | 49 | 36 | 9 | 5 | 2 |
| I had rapidly switching thoughts. | 16 | 27 | 22 | 27 | 8 |
| I had superficial thoughts. | 8 | 27 | 25 | 37 | 3 |
| I thought about the past. | 35 | 38 | 11 | 15 | 2 |
| I thought about the present. | 9 | 10 | 16 | 55 | 11 |
| I thought about the future. | 20 | 26 | 20 | 30 | 5 |
| I had deep thoughts. | 26 | 40 | 21 | 11 | 2 |
| I thought about nothing. | 42 | 29 | 16 | 10 | 3 |
| I had difficulty holding on to my thoughts. | 20 | 37 | 23 | 18 | 2 |
| I thought about people I like. | 23 | 25 | 18 | 27 | 7 |
| I thought in images. | 21 | 19 | 16 | 35 | 9 |
| I thought in words. | 12 | 22 | 16 | 39 | 11 |
| I thought about things I need to do. | 18 | 22 | 15 | 35 | 9 |
| I was conscious of my body. | 6 | 11 | 14 | 51 | 18 |
| I thought about the sounds around me. | 14 | 24 | 20 | 32 | 10 |
| I thought about the odors around me. | 42 | 41 | 10 | 5 | 1 |
| I thought about my heartbeat. | 40 | 36 | 8 | 12 | 3 |
| I thought about my breathing. | 22 | 20 | 13 | 30 | 15 |
| I placed myself in other peoples shoes. | 43 | 34 | 13 | 9 | 1 |
| I had negative feelings. | 47 | 32 | 13 | 7 | 1 |
| I had my thoughts under control. | 5 | 16 | 26 | 44 | 10 |
| I felt ill. | 73 | 18 | 6 | 2 | 0 |
| I felt pain. | 53 | 26 | 7 | 11 | 2 |
| I felt motivated to participate. | 0 | 0 | 20 | 60 | 20 |
| I have difficulty remembering my thoughts. | 39 | 47 | 14 | 0 | 0 |
| I have difficulty remembering my feelings. | 39 | 49 | 12 | 0 | 0 |
| I had my eyes closed. | 0 | 0 | 0 | 33 | 67 |
| I was able to rate the statements. | 0 | 0 | 0 | 60 | 40 |
